# Three-Dimensional Molecular Modeling of a Diverse Range of SC Clan Serine Proteases

**DOI:** 10.1155/2012/580965

**Published:** 2012-11-19

**Authors:** Aparna Laskar, Aniruddha Chatterjee, Somnath Chatterjee, Euan J. Rodger

**Affiliations:** ^1^Infectious Diseases and Immunology Division, CSIR-Indian Institute of Chemical Biology, West Bengal, Kolkata 700032, India; ^2^Department of Pathology, Dunedin School of Medicine, University of Otago, P.O. Box 913, Dunedin 9054, New Zealand; ^3^National Research Centre for Growth and Development, University of Auckland, Auckland 1142, New Zealand

## Abstract

Serine proteases are involved in a variety of biological processes and are classified into clans sharing structural homology. Although various three-dimensional structures of SC clan proteases have been experimentally determined, they are mostly bacterial and animal proteases, with some from archaea, plants, and fungi, and as yet no structures have been determined for protozoa. To bridge this gap, we have used molecular modeling techniques to investigate the structural properties of different SC clan serine proteases from a diverse range of taxa. Either SWISS-MODEL was used for homology-based structure prediction or the LOOPP server was used for threading-based structure prediction. The predicted models were refined using Insight II and SCRWL and validated against experimental structures. Investigation of secondary structures and electrostatic surface potential was performed using MOLMOL. The structural geometry of the catalytic core shows clear deviations between taxa, but the relative positions of the catalytic triad residues were conserved. Evolutionary divergence was also exhibited by large variation in secondary structure features outside the core, differences in overall amino acid distribution, and unique surface electrostatic potential patterns between species. Encompassing a wide range of taxa, our structural analysis provides an evolutionary perspective on SC clan serine proteases.

## 1. Introduction

Serine proteases account for over a third of all known proteolytic enzymes and are involved in a range of physiological processes including digestion, immunity, blood clotting, fibrinolysis, reproduction, and protein folding [1]. The proteolytic mechanism of these proteases involves nucleophilic attack of the carbonyl atom of the substrate peptide bond by a catalytic serine (Ser) residue in the active site of the enzyme. In addition to the nucleophilic Ser residue, this reaction is dependent on other critical amino acids in the catalytic site such as an Aspartate (Asp) and a Histidine (His) that together form what is referred to as the catalytic triad (or a dyad in some cases) [2]. The presence of this catalytic triad in at least four distinct protein folds indicates the same mechanism evolved four separate times during evolution [3].

The MEROPS classification system (http://merops.sanger.ac.uk/) has grouped proteases into families according to statistically significant similarities in the amino acid sequence. These protease families are further grouped into clans that have dissimilar amino acid sequences but typically have structural homology and/or the same linear order of catalytic triad residues [4]. The SC clan of serine proteases is widely distributed across all taxa, and in contrast to other clans, it includes both endopeptidases and exopeptidases. At the core of all SC clan proteases is an *α*/*β* hydrolase fold, which typically consists of an eight-stranded *β*-sheet flanked by two or more *α*-helices. The *α*-helices contribute to substrate specificity, and the curvature of the *β*-sheet may also affect interactions with the substrate [1]. The *α*/*β* hydrolase fold is a common hydrolytic enzyme structure and is found in many other enzymes such as lipases, peroxidases, and esterases [5]. The SC clan has the same classical serine protease catalytic triad residue formation as clans SB and PA, but with the amino acid sequence order of Ser, Asp, and His. Typically, these residues are confined to the C-terminal region within about 130 residues. The proteolytic mechanism is initiated by the nucleophilic Ser158 (standard serine carboxypeptidase 2 *CBP2* numbering) hydroxyl group transferring a proton to the carbonyl of the peptide substrate. This reaction is catalyzed by the His413 acting as a general base, which is thought to be supported by a hydrogen bond to Asp361. The resulting tetrahedral intermediate breaks down to an acylenzyme intermediate, followed by the formation of a second tetrahedral intermediate. With the protonation of Ser158 by His413, the second tetrahedral intermediate breaks down and the cleaved substrate is released [2].

The five main families in this clan have distinct specificities and have different peptidase activities as represented by the archetypes prolyl oligopeptidase (S9 family), carboxypeptidase Y (S10 family), Xaa-Pro dipeptidyl-peptidase (S15 family), lysosomal Pro-Xaa carboxypeptidase (S28 family), and prolyl aminopeptidase (S33 family).

Because of their abundance and biological significance, the S9 serine protease family has been the most intensively studied. The proteases of this family are up to three times larger than their classic serine protease counterparts, trypsin and subtilisin (25–30 kDa). Many members hydrolyze the peptide bond on the C-terminal side of proline, but the exceptions include oligopeptidase B, which recognizes arginine or lysine, and acylaminoacyl peptidase, which is a cytoplasmic omega-peptidase that releases an N-acylated amino acid [6]. Notably, the central tunnel of an unusual N-terminal *β*-propeller domain covers the catalytic site and selectively restricts access to oligopeptides of approximately 30 amino acids in length [7]. The S9 family appears to be important in the processing and degradation of peptide hormones, and, therefore, these proteases are important targets of drug design [8]. In humans, prolyl oligopeptidase is involved in several neurological conditions and control of blood pressure [9–11], dipeptidyl peptidase 4 in type 2 diabetes and cancer [12, 13], and acylaminoacyl peptidase in small-cell lung and renal cancer [14, 15]. Both prolyl oligopeptidase and oligopeptidase B seem to facilitate the virulence of protozoan parasites such as *Trypanosoma cruzi* and *Trypanosoma brucei*, which result in the trypanosome infections Chagas disease and sleeping sickness, respectively [16, 17]. Dipeptidyl peptidase 4 contributes to the pathogenicity of *Porphyromonas gingivalis*, the gram-negative bacteria associated with periodontitis [18].

Proteases in the S10 family are serine carboxypeptidases, which cleave C-terminal peptide bonds. They generally prefer hydrophobic amino acids but exhibit broad substrate specificity. In contrast with most other serine proteases, which are typically active at neutral/alkaline pH, family S10 proteases maintain catalytic activity in an acidic environment [19]. This family mostly contributes to proteolytic degradation and protein processing within specific cellular compartments such as vacuoles in fungi and plants (carboxypeptidase Y) and lysosomes in animals (serine carboxypeptidase A) [20, 21]. Members of the S15 family selectively cleave Xaa-Pro, in which Xaa is an N-terminal amino acid. In *Lactobacillus helveticus*, which is used for commercial cheese-making, Xaa-Pro dipeptidyl-peptidase is involved in the casein-degradation pathway, providing essential amino acids for the bacteria [22]. The S28 proteases are a distinct family of eukaryotic carboxypeptidases that selectively cleave a Pro-Xaa bond, in which Xaa is a C-terminal amino acid. The human lysosomal Pro-Xaa carboxypeptidase (*PRCP*) is thought to be involved in regulating blood pressure by inactivating angiotensin II [23]. Dipeptidyl peptidase 2, which has a similar substrate specificity to dipeptidyl peptidase 4 of the S9 family, is essential for maintaining lymphocytes and fibroblasts in a quiescent state [24]. Proteases in the S33 family are prolyl aminopeptidases, which preferentially cleave an N-terminal proline residue peptide bond. Many of the bacteria and fungi that produce prolyl aminopeptidases are pathogenic and have therefore been proposed as a viable drug target [25].

## 2. Material and Methods

Structural data for 3 bacterial, 2 archaeal, 2 fungal, and 4 animal SC clan serine protease structures (Table 1) were obtained from the Protein Data Bank (PDB, http://www.rcsb.org/pdb/). Our inhouse modeling software package MODELYN [26] was developed to perform customized molecular editing and *in silico* structural analysis. It has a set of powerful menus for batch processing commands leading to automated implementation of complicated tasks, including complete model building based on sequence homology and batch processing of replacement mutations. ANALYN [26] is an ancillary protein sequence analysis program that assists MODELYN by analyzing homologous sequences and formulating the strategy for model building. In addition to the experimental structures, amino acid sequences of SC serine proteases (Table 1) for 1 plant (*Arabidopsis thaliana*) and 1 protozoan (*Plasmodium falciparum*) were obtained from the MEROPS protease database (http://merops.sanger.ac.uk/) in FASTA format [27]. These sequences were initially submitted to SWISS-MODEL for homology-based structure prediction [28]. If a sequence had less than 25% sequence similarity with known experimental structures, these sequences were then submitted to the LOOPP server [29] for threading-based structure prediction as previously described [30, 31]. This analysis reported a ranked list of possible structure predictions for each of the protease sequences, including match scores, sequence identity (%), and the extent of sequence coverage (%). Predicted structures were superposed with respect to a selected set of C*α* atoms on the structure with the highest match score, and a suitable starting scaffold was determined using MODELYN. Root mean square deviation (RMSD) values helped to identify the common segments, corresponding to the structurally conserved regions. The initial structures were refined using the DISCOVER and ANALYSIS modules within the software package Insight II [32] through energy minimization and molecular dynamics. The side chains were regenerated using SCRWL [33], and the overall structure was energy minimized. The SCWRL software package was used for prediction of protein side-chains of a fixed backbone, using graph theory to solve the combinatorial problem (details of the structure refinement are given in the Supplementary Material available online at doi:10.1155/2012/580965). PROCHECK was used to check the distribution of *φ*-*ψ* dihedral angles and identify Ramachandran outliers [34]. The CHARMM module within Insight II was used to apply dihedral constraints in these segments. MOLPROBITY [35] and MODELYN were used to validate the structural models against experimental structure data. MOLPROBITY provides all-atom contact analysis and gives quantitative information on the steric interactions (H-bond and van der Waals contacts) at the interfaces between components. This program is widely used for quality validation of three-dimensional (3D) protein structures by measuring deviations of bond lengths, bond angles from standard values, overall atom clashscores, and rotamer outliers. MODELYN was used to analyze other structural parameters, including the distance between C*α* atoms of the catalytic triad. Verify3D [36], ProSA [37], and ERRAT [38] were also used to further assess the quality of the protease models. Verify3D analyzes the compatibility of the model against its own amino acid sequence. The Verify3D score (the sum of scores for individual residues using a 21-residue sliding window) is normalized to the length of the sequence: log_2_(Verify3D score/L^2^) [39]. ProSA calculates an overall quality score (Z score) of a model in comparison to a range of characteristics expected for native protein structures. ERRAT analyzes the statistics of nonbonded interactions between different atom types (9-residue sliding window) and provides an overall quality factor that is expressed as the percentage of the protein for which the calculated error value falls below the 95% threshold. The ribbon structure and electrostatic potential surface of the structures were determined by MOLMOL [40]. To determine sequence conservation between species, ClustalW [41] was used for multiple sequence alignment. For each sequence, PEPSTATS [42] was used to determine the molar percentage of each amino acid physicochemical class. A flowchart of the modeling and structure refinement strategy has been included as Supplementary Figure S1.

## 3. Results

### 3.1. Modeling of Protease Structures

The plant protease from *A. thaliana *had significant homology with proteases of known experimental structure for successful structure prediction using SWISS-MODEL. The homology model was essentially built on the structures 2BKL, 1YR2, 1VZ2 (prolyl oligopeptidases from *Myxococcus xanthus*, *Novosphingobium capsulatum*, and *Sus scrofa* resp.), and 1QGS (an spsA glycosyltransferase from *Bacillus subtilis*), with sequence identity ranging from 30% to 34% (Table 2). Homology-based structure prediction for the *P. falciparum* protease was unsuccessful due to insufficient sequence similarity with known experimental structures. The amino acid sequence was then submitted to the LOOPP server for threading-based structure prediction, which yielded a list of 14 different PDB experimental structures that matched the protease sequence. The matching structures showed good confidence scores ranging from 2.7 to 3.5, sequence identity ranging from 13% to 19%, with best length coverage between 86% and 100% (Table 3). The matched structures were superposed with respect to a selected set of *P. falciparum* protease C*α* atoms (43% superposition), with the structure 1U2E (an MhpC C–C bond hydrolase from *Escherichia coli*) having the best score of 3.5 (RMSD values were between 0.332 and 0.564 Å, which helped to identify common segments corresponding to structurally conserved regions). From these superposed structures, the variable loop regions were identified on the starting scaffold derived from 1U2E. Structural refinement of the two models using Insight II and SCRWL is provided in detail as Supplementary Material (additional file 1). The overall backbone conformations of the predicted structures were measured, and Ramachandran outliers were corrected for by applying dihedral constraints in these segments (Table 4). The general structural parameters and the overall quality of the final refined model were compared to experimental structure data (Table 5). The physical parameters were comparable between the experimental and predicted structures. The good scores provided by Verify3D, ProSA, and ERRAT further validated the overall quality of the refined models from *A. thaliana* (PMDB: PM0078228) and *P. falciparum* (PMDB: PM0078229).

### 3.2. Catalytic Core Geometry

**Figure 1:**
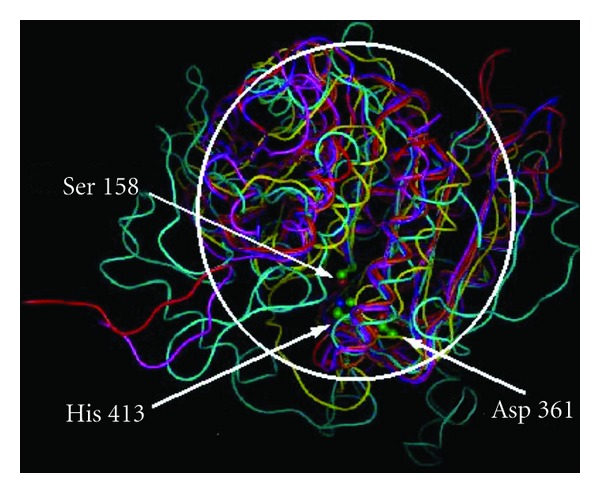
Superposed structures of X-ray and modeled structures of the selected proteases of the SC clan. Structures of the plant (PM0078228, *Arabidopsis thaliana*, purple) and protozoan (PM0078229, *Plasmodium falciparum*, yellow) SC proteases were superposed with the animal (1QFS, *Sus scrofa*, red), archaeon (1VE7, *Aeropyrum pernix*, magenta), fungal (1WPX, *Saccharomyces cerevisiae*, cyan), and bacterial (2BKL, *Myxococcus xanthus*, orange) X-ray structures. The catalytic triad residues (Ser, Asp, His; *CBP2* residue numbering used as a standard reference) are shown in ball and stick models, and the core regions of the structures are indicated by the white circle.

**Figure 2:**
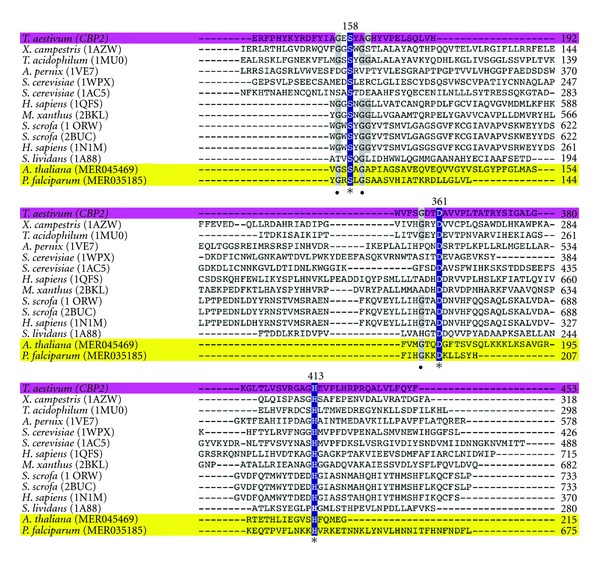
Multiple amino acid sequence alignment of SC serine proteases. ClustalW was used to align amino acid sequences of SC serine proteases for which their structures were determined experimentally or predicted computationally (highlighted in yellow). Wheat serine carboxypeptidase 2 (*CBP2*, highlighted in magenta) is used as a standard reference for residue numbering. Only the regions showing the conserved catalytic residues Ser (S), Asp (D), and His (H) are shown (highlighted in blue). Other residues showing medium (**•**) conservation are highlighted in gray.

Superposition of the *A. thaliana* and *P. falciparum* proteases on the representative 1U2E protease structure found that 17% to 69% of the C*α* atoms superposed with an RMSD below 1.1 Å (Table 6). In comparison, X-ray protease structures had 10% to 28% of the C*α* atoms superposed with an RMSD below 1.5 Å (Table 6). The superposed structures have a common core structure with large variation in loops outside the core (Figure 1). The C*α* atom distances of Asp to His, His to Ser, and Asp to Ser averaged over the experimentally determined structures were 4.6 ± 0.03, 7.9 ± 0.06, and 10.5 ± 0.04 Å, respectively (Table 6). The small standard deviations (SDs) indicated that the structural environment around the catalytic triad was highly conserved. Averaged over the predicted structures, the C*α* atom distances between the catalytic triad residues were 4.6 ± 0.01, 8.2 ± 0.01, and 10.9 ± 0.25 Å, respectively, in good agreement with the values averaged over the experimental structures. Multiple sequence alignment (Figure 2) confirmed sequence conservation of the catalytic triad residues at Ser158 Asp361 His413 (serine carboxypeptidase 2 numbering). Among the sequences analyzed, the highly conserved amino acids Gly156 and Gly160 had the occupancy percentage of 70% and 77%, respectively, which has been previously described [43]. In addition, Gly161, Asp315, Val317, and Gly343 were all highly conserved with an occupancy percentage of 75% in the S9 family member sequences analyzed. As confirmed in other serine proteases, such residues may confer stabilization of the catalytic site via a hydrogen-bonding interaction [44, 45]. By incorporating an evolutionarily diverse range of SC serine proteases, our analysis indicates that although the core structures deviated considerably during evolution, the relative positions of the catalytic triad C*α* atoms maintained very close relative distances and were potentially stabilized by other highly conserved residues.

### 3.3. Structural Analysis

**Figure 3:**
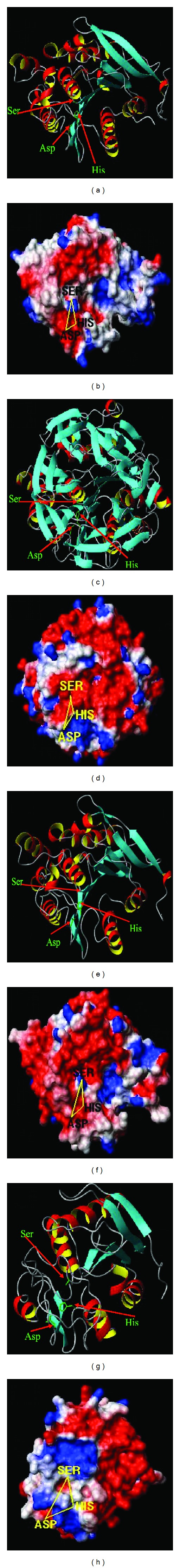
Representative X-ray SC protease structures and modeled SC protease structures from *Arabidopsis thaliana* and *Plasmodium falciparum.* Ribbon models of *S. scrofa,* 1QFS (a), *A. pernix,* 1VE7 (c), *A. thaliana* (e), and *P. falciparum* (g) SC protease structures show *β*-sheets with an arrow directed to the C-terminus (light blue), *α*-helices (red and yellow), turn/loops (gray), and catalytic triad residue side chains (green sticks). Surface electrostatic potential models of *S. scrofa,* 1QFS (b), *A. pernix,* 1VE7 (d), *A. thaliana,* PM0078228 (f), and *P. falciparum,* PM0078229 (h) SC protease structures show electronegative (red), electropositive (blue), and electroneutral (white) amino acid side chains. Electrostatic potential thresholds: −1.4 kT/e < 0.0 kT/e < +1.4 kT/e (red → white → blue).

The catalytic core of all SC clan proteases bears an *α*/*β* hydrolase fold, which typically consists of a *β*-sheet flanked by two or more *α*-helices. Figures 3(a) and 3(b) are representative X-ray structures of an animal SC protease (1QFS, prolyl oligopeptidase from *Sus scrofa*), comprising 8 *β*-sheets and 12 *α*-helices. Figures 3(c) and 3(d) are representative X-ray structures of an archaeon SC protease (1VE7, acylpeptidase hydrolase from *Aeropyrum pernix*), comprising 30 *β*-sheets and 14 *α*-helices. The *A. thaliana* SC protease model had 8 *β*-sheets and 12 *α*-helices, with Ser120, Asp176, and His198 in separate turn/coil structures (Figure 3(e)). The electrostatic potentials around the Asp and His catalytic residues were mostly electronegative, and there was a patch of electropositive potential around the Ser residue of the catalytic triad (Figure 3(f)). The electronegative region in the catalytic site of the modeled protease could facilitate specificity by favoring positively charged C-terminal amino acid side chains at specific sites within the binding pocket. The *A. thaliana* protease had a higher proportion (>SD of the mean) of aliphatic residues (32%, molar percentage), compared to other species (see Table S1), which could influence stability of the enzyme at a wide range of temperatures [46]. According to MEROPS annotation (MER045469), this protease has been assigned to the S9 family, but it has an unknown function. Our homology model was essentially built on the structures 2BKL, 1YR2, and 1VZ2, which are prolyl oligopeptidases (S9 family) from *Myxococcus xanthus*, *Novosphingobium capsulatum*, and *Sus scrofa,* respectively. There have been 23 genes encoding prolyl oligopeptidase-like proteins identified in *A. thaliana* [47]. Although the function of most of these is unknown, there is some evidence that prolyl oligopeptidase is involved in seed development [48]. *A. thaliana* is a highly studied model organism, and mutational analysis of this protease would be useful to explore these features.

The protease model from *P. falciparum* had 7 *β*-sheets and 7 *α*-helices, with Ser124, Asp188, and His217 in separate turn/coil structures (Figure 3(g)). The surface electrostatic potentials around the catalytic site were very different to those of other clan members studied, with large patches of electropositive and electroneutral regions around the catalytic triad residues (Figure 3(h)). The largely electropositive catalytic site of this modeled protease suggests it favors a negatively charged substrate. The largely electroneutral regions possibly relax the stringency of the substrate binding, allowing for a number of different protein substrates. In comparison with the other species analyzed (see Table S1), the *P. falciparum* protease had a higher proportion (>SD of the mean) of polar residues (60%, molar percentage) and basic amino acids (16%), which indicates it could favor a more hydrophilic environment. Like the modeled protease from *A. thaliana*, this protease (MER035185) has also been assigned to the S9 family. Although the function of this protease is not known, it is of interest that both prolyl oligopeptidase and oligopeptidase B of the S9 family appear to facilitate the virulence of other protozoan parasites such as *Trypanosoma cruzi* and *Trypanosoma brucei* [16, 17]. Further investigation of substrate specificity and other properties contributing to it would be beneficial for functional analysis of this protease, as it could be a potential target for rational antimalarial drug design.

The following predicted structures are available in the Protein Model Database (PMDB) (http://mi.caspur.it/PMDB/):

1. SC serine protease from *Arabidopsis thaliana* (PMDB ID: PM0078228),
2. SC serine protease from *Plasmodium falciparum* (PMDB ID: PM0078229).

## 4. Conclusion

In conjunction with 11 experimentally determined 3D protein structures, our analysis of predicted structures from a plant and a protozoan encompassed an evolutionarily diverse range of SC clan proteases. The structural geometry of the catalytic core clearly deviated considerably during evolution, but the relative positions of the catalytic triad residues were conserved, and other highly conserved residues possibly provide stabilization of the core. Evolutionary divergence was also exhibited by large variation in secondary structure features outside the core, differences in overall amino acid distribution, and unique surface electrostatic potential patterns between species. These features are probably associated with environmental adaptation, subcellular localisation, and the diverse functions of the different protease orthologs. The modeled proteases from *A. thaliana* and *P. falciparum* appear to be prolyl oligopeptidases of the S9 family. Evidence indicates that prolyl oligopeptidase is involved in plant seed development [48] and facilitates the virulence of protozoan parasites [16, 17]. Further structural investigation of these proteases would be useful for protein engineering strategies and for rational drug design in the case of the *P. falciparum* protease.

## Supplementary Material

The Supplementary Material provides a detailed methodology of protease modeling structural refinement, a comparison of protease amino acid composition based on physico-chemical properties and a flowchart of protease modeling and structure refinement.

## Figures and Tables

**Table 1:** Experimental structures and predicted structures of SC serine proteases across different taxa.

Species	Structure	MEROPS ID
Bacteria		
*Xanthomonas campestris *	PDB: 1AZW	MER002678
*Myxococcus xanthus *	PDB: 2BKL	MER005694
*Streptomyces lividans *	PDB: 1A88	MER026339
Archaea		
*Thermoplasma acidophilum *	PDB: 1MU0	MER003537
*Aeropyrum pernix *	PDB: 1VE7	MER005807
Fungi		
*Saccharomyces cerevisiae *	PDB: 1AC5	MER000413
*Saccharomyces cerevisiae *	PDB: 1WPX	MER002010
Animalia		
*Homo sapiens *	PDB: 1N1M	MER000401
	PDB: 1QFS	MER000392
*Sus scrofa *	PDB: 1ORW	MER028372
	PDB: 2BUC	MER028372
Plantae		
*Arabidopsis thaliana *	**PMDB: PM0078228**	MER045469
Protozoa		
*Plasmodium falciparum *	**PMDB: PM0078229**	MER035185

**Table 2:** SWISS-MODEL homology results of *Arabidopsis* *thaliana* SC serine protease target sequence with known PDB structures.

PDB ID	Resolution (Å)	*R*-value	Score (bits)	Expect value	AA identity (%)
2BKLB	1.50	0.161	47.4	9 × 10^−7^	30
2BKLA	1.50	0.161	47.4	9 × 10^−7^	30
1YR2A	1.80	0.162	40.8	9 × 10^−6^	34
1QFSA	2.00	0.201	39.7	9 × 10^−5^	30
1VZ2A	2.20	0.165	39.7	2 × 10^−2^	30

**Table 3:** LOOPP server results for secondary structure matches of *Plasmodium falciparum* SC serine protease target sequence with known PDB structures.

PDB ID	Secondary structure			Score	Sequence identity (%)	Length (%)
	Helical structure (%)	Extended (%)	Loops/other (%)			
Target	36.20	19.00	44.80	—	—	—
1U2E	39.44	18.31	42.25	3.547	19.46	100.00
1J1I	41.80	19.53	38.67	3.484	14.88	97.29
1UKS	40.15	17.84	42.01	3.353	18.35	98.64
1C4X	40.71	16.43	42.86	3.294	14.68	98.64
1CQW	32.98	18.85	48.17	3.207	15.67	85.97
1BN6	32.46	19.37	48.17	3.205	15.67	85.97
1MJS	32.53	18.69	48.79	3.158	18.64	99.55
1BN7	32.98	18.47	43.55	3.042	17.73	99.55
1B6G	33.44	14.75	51.80	3.036	15.84	100.00
1FJ2	29.65	19.91	50.44	2.999	19.71	94.12
1JJF	32.40	20.40	47.20	2.997	16.29	100.00
1EHY	38.43	17.08	44.48	2.937	14.55	99.55
1IMJ	30.73	21.95	47.32	2.933	12.79	92.31
1A85	42.28	15.81	41.91	2.688	17.06	95.48

**Table 4:** Backbone refinement of the modeled SC proteases from *Arabidopsis* *thaliana* and *Plasmodium* *falciparum*.

	*φ*-*ψ* distribution in the regions of Ramachandran plot			
Structural model	Number of residues (percentage)			
	Most favoured	Additionally allowed	Generously allowed	Disallowed
*A. thaliana *				
Before backbone refinement	190 (78.8%)	43 (17.8%)	4 (1.7%)	4 (1.7%)
After backbone refinement	194 (80.8%)	46 (19.2%)	0 (0.0%)	0 (0.0%)
*P. falciparum *				
Before backbone refinement	132 (65.7%)	53 (26.4%)	10 (5.0%)	6 (3.0%)
After backbone refinement	129 (65.5%)	68 (34.5%)	0 (0.0%)	0 (0.0%)

**Table 5:** Structural validation of the modeled SC proteases from *Arabidopsis thaliana *and *Plasmodium falciparum. *

Structural model	All-atom clashscore (No/1000 atoms)	Rotamer outliers (%)	RMSD of bond length (Å)	RMSD of bond angle (degree)
X-ray structure (1YR2)	3.76	2.40	0.240	2.76
Homology model of *A. thaliana* protease	5.37	2.14	0.270	3.70
X-ray structure (1U2E)	23.50	9.62	0.013	2.27
Threading model of *P. falciparum* protease	11.30	4.50	0.028	3.26

	Average Verify3D-1D score	Normalized 3D profile score (log_2_(Verify3D/L^2^)	ProSA *Z*-score	ERRAT quality factor (%)

X-ray structure (1YR2)	0.46	−10.53	−9.68	95.2
Homology model of *A. thaliana* protease	0.34	−9.66	−7.55	88.9
X-ray structure (1U2E)	0.42	−10.86	−9.88	93.7
Threading model of *P. falciparum* protease	0.29	−9.58	−4.53	75.5

**Table 6:** Structural parameters of experimentally determined and predicted 3D structures of SC serine proteases.

ID	Taxa	Species	Superposed of AA %	RMSD Å	Distances between the catalytic triad Å		
					(D-H)	(H-S)	(S-D)
1AZW	Bacteria	*X. campestris *	10.20	1.330	4.4	7.8	10.5
2BKL	Bacteria	*M. xanthus *	27.60	0.726	4.6	8.3	10.6
1A88	Bacteria	*S. lividans *	12.30	1.080	4.6	7.9	10.6
1VE7	Archaea	*A. pernix *	15.76	1.083	4.7	8.4	10.4
1MU0	Archaea	*T. acidophilum *	17.00	1.013	4.3	7.8	10.1
1AC5	Fungi	*S. cerevisiae *	17.86	1.441	4.8	7.7	10.8
1WPX	Fungi	*S. cerevisiae *	12.30	1.168	4.6	7.4	10.4
1N1M	Animalia	*H. sapiens *	16.29	1.163	4.8	7.7	10.7
1QFS	Animalia	*S. scrofa *	100	0.000	4.6	8.1	10.5
1ORW	Animalia	*S. scrofa *	18.29	1.092	4.8	7.7	10.7
2BUC	Animalia	*S. scrofa *	17.15	1.108	4.8	7.7	10.7

Mean ± SD of the C*α* distances between the triad residues					4.6 ± 0.03	7.9 ± 0.06	10.5 ± 0.04

**PM0078228**	Plantae	*A. thaliana *	16.7	1.021	4.5	8.2	11.2
**PM0078229**	Protozoa	*P. falciparum *	69.3	0.686	4.6	8.1	10.5

Mean ± SD of the C*α* distances between the triad residues					4.6 ± 0.01	8.2 ± 0.01	10.9 ± 0.25
