# Supplementary material for: Three-Dimensional Molecular Modeling of a Diverse Range of SC Clan Serine Proteases
Source: Mol Biol Int. 2012 Nov 19;2012:580965. doi: 10.1155/2012/580965 (PMC3507156; doi:10.1155/2012/580965)
Supplement: Supplementary file 1 — The Supplementary Material provides a detailed methodology of protease modeling structural refinement, a comparison of protease amino acid composition based on physico-chemical properties and a flowchart of protease modeling and structure refinement. [file 580965.f1.docx]

# Additional files

### Additional file 1

**Structural Refinement of Modeled SC proteases**

The starting structures were refined using the Insight II equipped with DISCOVER as the energy minimization and molecular dynamics module. Structural optimization involved energy minimization (100 steps each of steepest descent and conjugate gradient methods) using cff91 force-field followed by dynamics simulations. A typical dynamics run consisted of 10000 steps of one femto-second (10 picoseconds) after 1000 steps of equilibration with a conformational sampling of 1 in 10 steps at 300K. However, dynamics simulation of 100 picoseconds was also applied to certain bigger loops for proper regularization. At the end of the dynamics simulation, the conformation with lowest potential energy was picked for the next cycle of refinement using the ANALYSIS module of Insight II. This combination of minimization and dynamics were repeated until satisfactory conformational parameters were obtained. Each loop was separately regularized applying position constraints to the rest of the atoms of the protein, which were 2 amino acids away from the desired loop by energy minimization and molecular dynamics followed by evaluation of the structural parameters. SCWRL was used to regenerate the sidechains of the modeled proteases. The final structures were energy minimized 100 steps each with steepest descent and conjugate gradient methods keeping all the atoms of the protein free.

**Table S1. Relative comparison of SC serine protease amino acid composition based on physico-chemical properties**

| **ID** | **Small (%)** | **Aliphatic (%)** | **Aromatic (%)** | **Nonpolar (%)** | **Polar (%)** | **Charged (%)** | **Basic (%)** | **Acidic (%)** |
| --- | --- | --- | --- | --- | --- | --- | --- | --- |
|  |  |  |  |  |  |  |  |  |
| **All residues** | | | | | | | | |
| *CBP2* | 56 | 27 | 16 | 57 | 43 | 21 | 11 | 10 |
| 1AZW | 51 | 30 | 17 | 57 | 43 | 28 | 15 | 13 |
| 1MU0 | 44 | 27 | 14 | 54 | 46 | 30 | 15 | 15 |
| 1VE7 | 54 | 32 | 9 | 59 | 41 | 25 | 13 | 13 |
| 1WPX | 53 | 27 | 14 | 55 | 45 | 24 | 10 | 14 |
| 1AC5 | 52 | 22 | 11 | 44 | 56 | 32 | 12 | 20 |
| 1QFS | 48 | 26 | 16 | 55 | 45 | 27 | 13 | 13 |
| 2BKL | 54 | 29 | 14 | 56 | 44 | 27 | 15 | 13 |
| 1ORW | 49 | 26 | 17 | 53 | 47 | 23 | 12 | 11 |
| 1BUC | 49 | 26 | 17 | 53 | 47 | 23 | 12 | 11 |
| 1N1M | 49 | 26 | 17 | 53 | 47 | 23 | 12 | 11 |
| 1A88 | 59 | 32 | 14 | 59 | 41 | 25 | 12 | 13 |
|  |  |  |  |  |  |  |  |  |
| MER045469 | 54 | 32 | 10 | 55 | 45 | 24 | 13 | 11 |
| MER035185 | 52 | 22 | 11 | 40 | 60 | 24 | 16 | 8 |
|  |  |  |  |  |  |  |  |  |
| Mean ± SD | 52 ± 4 | 27 ± 3 | 14 ± 3 | 53 ± 5 | 47 ± 5 | 25 ± 3 | 13 ± 2 | 13 ± 3 |
|  |  |  |  |  |  |  |  |  |

* Amino acid physico-chemical classes: small (Gly, Ala, Ser, Pro, Val, Thr, Cys), aliphatic (Ile, Leu, Val), aromatic (Phe, His, Trp, Tyr), non-polar (Ala, Cys, Phe, Gly, Ile, Leu, Met, Pro, Val, Trp, Tyr), polar (Asp, Glu, His, Lys, Asn, Gln, Arg, Ser, Thr), charged (Asp, Glu, His, Lys, Arg), basic (His, Lys, Arg), and acidic (Asp and Glu).


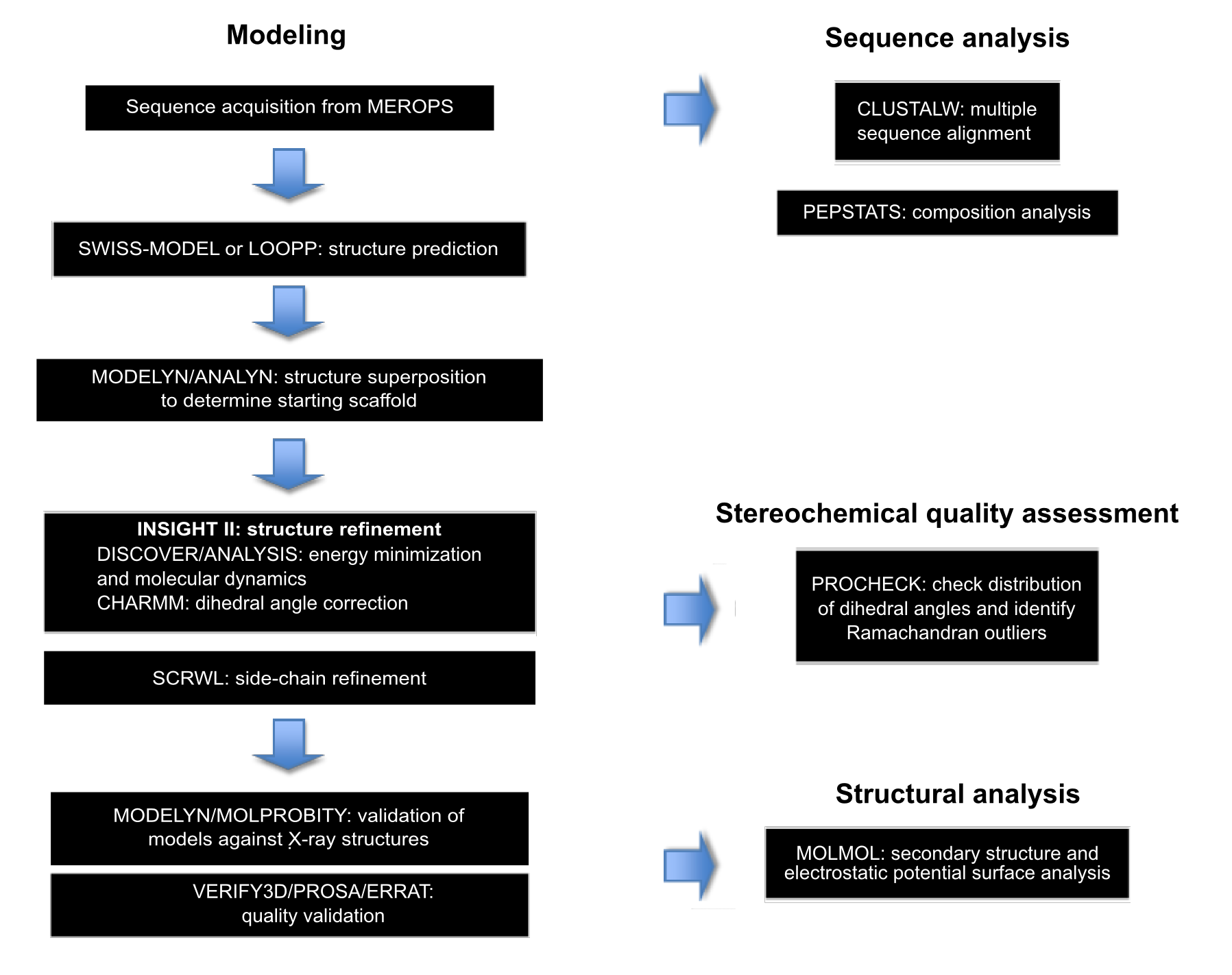


**Figure S1. Flowchart of modeling and structure refinement of SC clan proteases.**
